# Effects of Initial Values and Convergence Criterion in the Two-Parameter Logistic Model When Estimating the Latent Distribution in BILOG-MG 3

**DOI:** 10.1371/journal.pone.0140163

**Published:** 2015-10-09

**Authors:** Ingo W. Nader, Ulrich S. Tran, Martin Voracek

**Affiliations:** Department of Basic Psychological Research and Research Methods, School of Psychology, University of Vienna, Vienna, Austria; University of Amsterdam, NETHERLANDS

## Abstract

Parameters of the two-parameter logistic model are generally estimated via the expectation-maximization algorithm, which improves initial values for all parameters iteratively until convergence is reached. Effects of initial values are rarely discussed in item response theory (IRT), but initial values were recently found to affect item parameters when estimating the latent distribution with full non-parametric maximum likelihood. However, this method is rarely used in practice. Hence, the present study investigated effects of initial values on item parameter bias and on recovery of item characteristic curves in BILOG-MG 3, a widely used IRT software package. Results showed notable effects of initial values on item parameters. For tighter convergence criteria, effects of initial values decreased, but item parameter bias increased, and the recovery of the latent distribution worsened. For practical application, it is advised to use the BILOG default convergence criterion with appropriate initial values when estimating the latent distribution from data.

## Introduction

The two-parameter logistic model (2PL; see [1]) is widely used, especially in medical settings and in health care applications (e.g., [2]). In this model, the probability of a correct response π_*i*_ is determined by two item parameters that are related to the ability of a test-taker by the equation

$$\pi_{i}\left(z_{j}\right)=\frac{\text{exp}\left[a_{i}\left(z_{j}-b_{i}\right)\right]}{1+\text{exp}\left[a_{i}\left(z_{j}-b_{i}\right)\right]},$$ (1)

where *a*_*i*_ and *b*_*i*_ label the item discrimination and item difficulty parameter of item *i*, respectively, and *z*_*j*_ denotes the latent ability of testee *j*. These parameters are generally estimated by means of the maximum likelihood (ML) method. The latent ability is often assumed to have a normal distribution and only the parameters of this distribution are estimated (marginal maximum likelihood method, MML). However, the assumption of a normally distributed ability is hardly ever met in practice [3], which may result in increased estimation error [4], and more so for smaller samples [5].

To account for non-normality, it is possible to estimate also the shape (and not only the parameters) of the latent distribution from data. One widely used method for estimating the latent distribution non-parametrically is the empirical histogram (EH) method [6]. Here, the latent distribution is defined as a set of weights at prespecified positions (support or quadrature points) on the latent dimension. These weights correspond to the relative frequency of testees with a specific position on the latent dimension (i.e., the specific ability at the support point) and are estimated in the maximization step of the EM algorithm. Hence, item parameters and latent distribution are estimated simultaneously.

As the ML equations do not have simple algebraic solutions, the expectation-maximization (EM) algorithm [7] is usually used to obtain ML estimates for the parameters. In this algorithm, assumed initial values for each estimated parameter are improved iteratively until some convergence criterion is met. Effects of initial values on outcomes are discussed intensively in other applications of the EM algorithm (e.g., mixture models or cluster analysis; see [8,9]), but rarely in item response theory (IRT) models. Although Bock [10], in an unpublished conference paper, stated that effects of initial values are negligible in IRT, a recent study showed effects of initial values on item parameters of the 2PL in full non-parametric ML estimation, where the latent distribution is also estimated from data [11].

The present study investigated a more common method of parameter estimation. By means of a Monte Carlo simulation, the present study investigated effects of initial values and of the convergence criterion on parameters of the 2PL in BILOG-MG 3 [12], a widely used software package in IRT employing MML estimation as well as the EH method.

## Simulation Study

### 2.1 Experimental factors

The five factors manipulated in this simulation study were (1) initial values for item parameters, (2) initial values for the latent distribution, (3) true latent distribution, (4) sample size, and (5) tightness of convergence criterion. Initial values for item parameters included (1a) true parameter values (items of a 20-item mathematical achievement test that were used for generating item responses; condition *true*), (1b) BILOG-MG 3 default values (condition *default*), (1c) identical values for all item parameters (value 1 for all item discrimination parameters, value 0 for all item difficulties; condition *const*), and (1d) values generated by short runs from the estimation procedure (condition *shortrun*). BILOG default values are generated by calculating classical test theory estimators from the estimation sample [13]. Condition *true* was used as a reference, whereas condition *const* is used by some other software packages (e.g., the R package ltm; see [14]). The *shortrun* condition was inspired by [8] and was already used in [11], with good results regarding item parameter bias. Starting from random values, ten iterations of the estimation procedure are performed. This is repeated fifty times, and the resulting parameters with the highest loglikelihood were used as initial values for this condition.

Initial values for the latent distribution (weights of support points; Factor 2) were (2a) uniform distribution (equal weights for all support points; condition *uniform*), (2b) BILOG-MG 3 default values (i.e., weights from a normal distribution; condition *default*), (2c) weights estimated by means of classical test theory (condition *est*), and (2d) values generated by the shortrun procedure (condition *shortrun*). For condition *est*, a sum score was calculated for each testee, weighted by a classical test theory approximation of the item discrimination parameter [15],

$${\hat{a}}_{i}=1.7\frac{r_{bis}}{\sqrt{1-r_{bis}^{2}}},$$ (2)

with *r*_*bis*_ denoting the biserial correlation of item *i* with the total number of items solved. The relative frequencies of weighted sum scores corresponding to each support point of the latent distribution were used as weights. For all conditions, the positions of the support points were equally spaced on the latent dimension.

Two forms of (true) latent distributions (Factor 3) were used for generating the simulation data of testee’s ability values: (3a) standard normal distribution and (3b) skew-normal distribution [16]. For better comparability with the standard normal distribution, the skewed distribution was mean-centered and scaled to have a standard deviation of 1. The shape parameter was set to ten, resulting in an average skewness of about 0.96. This is appropriate for a wide range of ability distributions found in practice [3].

Factor 4 manipulated sample size (250, 500, and 1000 testees), and Factor 5 accommodated different settings for the convergence criterion, using values of 10^−2^ (BILOG-MG 3 default), 10^−3^, 10^−4^, and 10^−5^. For all simulations runs, ten support points were used.

Additionally, model calculations were also performed by means of standard MML estimation. Default values from BILOG (weights from a standard normal distribution) were used as an assumed latent distribution. Factors were identical to those used for EH estimation (but Factor 2 was excluded).

Different seeds were used for each combination of true latent distribution and sample size (Factors 3 and 4). For all other factors, identical seeds were used within each replication. Hence, comparison of different initial values (for item parameters and latent distribution; Factors 1 and 2) and different convergence criteria (Factor 5) was based upon identical samples.

### 2.2 Generation of item responses

Item parameters were taken from a 20-item mathematics achievement test that was used in previous related research [4,11]. Person ability values were drawn from a *N*(0,1) distribution or from a skew-normal distribution, depending on Factor 3. Using these parameters, the probability of a correct response was calculated according to Eq 1 and compared to a random number sampled from a uniform *U*(0,1) distribution. The item was scored as correct if the probability of the correct response exceeded the random number, and as incorrect otherwise. Random numbers were generated by means of the Mersenne twister algorithm [17] implemented in R 2.11.1 [18].

### 2.3 Simulation Runs

BILOG-MG 3 was used for estimation of item parameters. All other calculations were performed in R 2.11.1. In each combination of experimental factors, 100 replications were performed. The maximum number of cycles was increased because of the stricter convergence criteria used (10000 EM cycles, 10 Newton cycles). For all other parameters, BILOG default values were used.

### 2.4 Outcome measures

Accuracy of parameter recovery was measured by calculating each item parameter’s bias over all replications,

$$Bias\left(\theta_{i}\right)=\frac{1}{100}\sum_{k=1}^{100}\left({\hat{\theta }}_{ik}-\theta_{i}\right),$$ (3)

where ${\hat{\theta }}_{ik}$ is the item discrimination (${\hat{a}}_{ik}$) or difficulty (${\hat{b}}_{ik}$) parameter estimate of a single item *i* in replication *k*, respectively, and *θ*_*i*_ is the corresponding true parameter. Additionally to bias of single items, the accuracy of the complete test was assessed by averaging absolute values of Eq 3 across all test items (mean absolute bias, also referred to as test-level bias).

As different combinations of item parameters can produce rather similar item characteristic curves (ICCs; see [19]), it is important to investigate combined effects of both item parameter estimates on ICC recovery. Accuracy of ICC recovery was assessed by the area between true and estimated ICC in a [-10,+10] interval of the latent continuum,

$$Bias\left(ICC\right)=\int_{-10}^{+10}\left|\pi \left(z|{\hat{a}}_{i},{\hat{b}}_{i}\right)-\pi \left(z|a_{i},b_{i}\right)\right|\;dz.$$ (4)

Bias values for each item (Eq 4; solved by numerical integration, using adaptive quadrature in connection with extrapolation by Wynn’s Epsilon algorithm and Gauss–Kronrod quadrature in the basic step) were averaged across all test items to assess ICC recovery in the complete test.

Analysis of variance (ANOVA) models were calculated to assess effect sizes of all factors used in this study. In these ANOVAs, averaged values of bias in the 100 replications in each condition were used as data. Hence, resulting *p* values are too small because of the reduced residual variance, compared to the variation of bias values across all individual 100 replications per condition. At the same time, holding the effect size fixed, *p* values are mostly only a function of the number of sampling points. However, this number may be increased arbitrarily in simulation studies, decreasing *p* values. Therefore, we deemed *p* values not informative in the present context and do not report them. Instead, absolute η^2^ is used as effect size, denoting each factor’s relative contribution (in terms of explained variance) to the averaged test-level bias and ICC bias, respectively.

To compare the distributions estimated by means of the EH method and the (true) distributions of the person ability parameters which were used to generate the data, Earth Mover’s Distance [20] was applied. This method compares two distributions (histograms) in terms of the minimum cost of turning one distribution into the other. Hence, it is a distance measure with higher values indicating lower accordance of the compared distributions.

## Results

### 3.1 Effects of initial values on item parameters

Effects of initial values for item parameters in the EH method were negligible and only explained 1.2% vs. 0.8% of variance in test-level bias of discrimination vs. difficulty parameters with the default convergence criterion of 10^−2^. For tighter convergence criteria, these minor effects diminished even further (Table 1).

**Table 1:** Explained Variance for Bias of Item Discrimination Parameter, Item Difficulty Parameter, and ICC Recovery for the Empirical Histogram Method.

		η^2^			
	*df*	10^−2^	10^−3^	10^−4^	10^−5^
Item discrimination parameter					
Initial item parameters (*iip*)	3	.0120	.0047	.0008	.0000
Initial latent distribution (*ild*)	3	.2630	.0950	.0336	.0267
Sample size (*n*)	2	.5176	.7140	.7661	.7688
True latent distribution (*tld*)	1	.1334	.1255	.1727	.1711
*iip* x *ild*	9	.0100	.0025	.0001	.0000
*iip* x *n*	6	.0002	.0002	.0005	.0000
*iip* x *tld*	3	.0245	.0072	.0005	.0001
*ild* x *n*	6	.0145	.0064	.0012	.0002
*ild* x *tld*	3	.0081	.0063	.0006	.0003
*n* x *tld*	2	.0087	.0301	.0234	.0326
Residual	57	.0082	.0083	.0005	.0001
Item difficulty parameter					
Initial item parameters (*iip*)	3	.0084	.0037	.0005	.0000
Initial latent distribution (*ild*)	3	.3039	.1258	.0608	.0527
Sample size (*n*)	2	.5174	.7879	.8920	.9013
True latent distribution (*tld*)	1	.0321	.0148	.0174	.0188
*iip* x *ild*	9	.0105	.0025	.0001	.0000
*iip* x *n*	6	.0002	.0005	.0002	.0000
*iip* x *tld*	3	.0453	.0131	.0011	.0002
*ild* x *n*	6	.0205	.0088	.0017	.0006
*ild* x *tld*	3	.0275	.0115	.0023	.0012
*n* x *tld*	2	.0228	.0233	.0228	.0245
Residual	57	.0114	.0083	.0012	.0006
ICC bias					
Initial item parameters (*iip*)	3	.0009	.0005	.0001	.0000
Initial latent distribution (*ild*)	3	.0179	.0089	.0036	.0030
Sample size (*n*)	2	.9410	.9556	.9609	.9603
True latent distribution (*tld*)	1	.0318	.0275	.0303	.0312
*iip* x *ild*	9	.0006	.0002	.0000	.0000
*iip* x *n*	6	.0000	.0000	.0000	.0000
*iip* x *tld*	3	.0022	.0009	.0001	.0000
*ild* x *n*	6	.0009	.0004	.0001	.0000
*ild* x *tld*	3	.0008	.0006	.0001	.0000
*n* x *tld*	2	.0029	.0047	.0048	.0054
Residual	57	.0009	.0007	.0000	.0000

*Note*. Mean-centered aggregated values (i.e., bias in each condition) were used as data. Therefore, residual variance is reduced. Absolute effect sizes η^2^ should be interpreted as the respective factor’s relative contributions to the total amount of bias. To avoid overfit, models only included main effects and two-way interactions.

**Fig 1:**
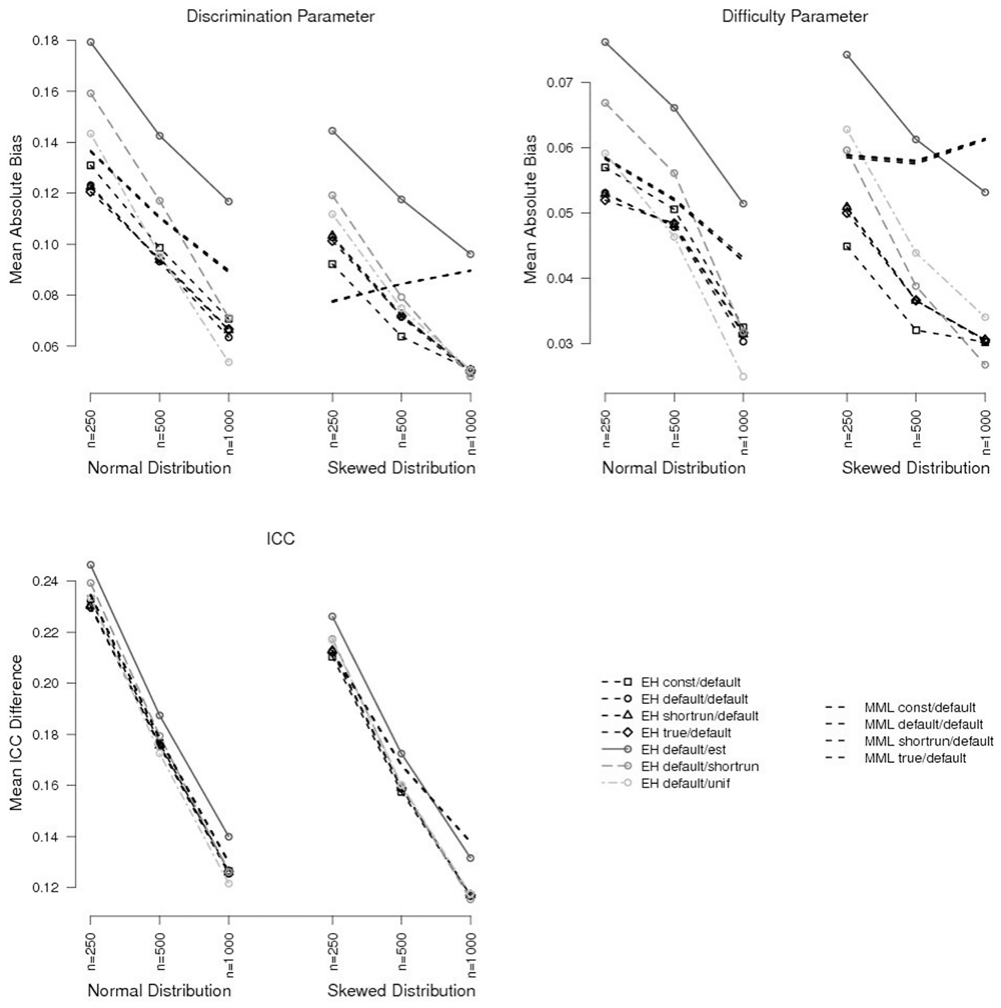
Test-level Bias and ICC Bias for Different Initial Values Using BILOG-MG 3 Default Convergence Criterion (10^−2^). For clarity, only selected conditions are shown.

Initial values for the latent distribution, on the other hand, had a stronger effect on test-level bias. For the default convergence criterion, they explained 26.3% vs. 30.4% of variance in test-level bias of discrimination vs. difficulty parameters. This was more than half of the amount of variance explained by sample size (which accounted for 51.8% vs. 51.7% of variance; Table 1). BILOG default values (condition *default*) performed well in all conditions, but were outperformed (lower values of test-level bias for discrimination and difficulty parameters) in large samples (*n* = 1000) by initial values from a uniform distribution (condition *unif*), when the true latent distribution was normal (Fig 1, top). When the true latent distribution was skewed, BILOG default values were the best choice (i.e., lowest values of test-level bias), except for large samples, where initial values from the shortrun procedure (condition *shortrun*) had lower values for difficulty parameter bias and slightly lower values for discrimination parameter bias.

**Fig 2:**
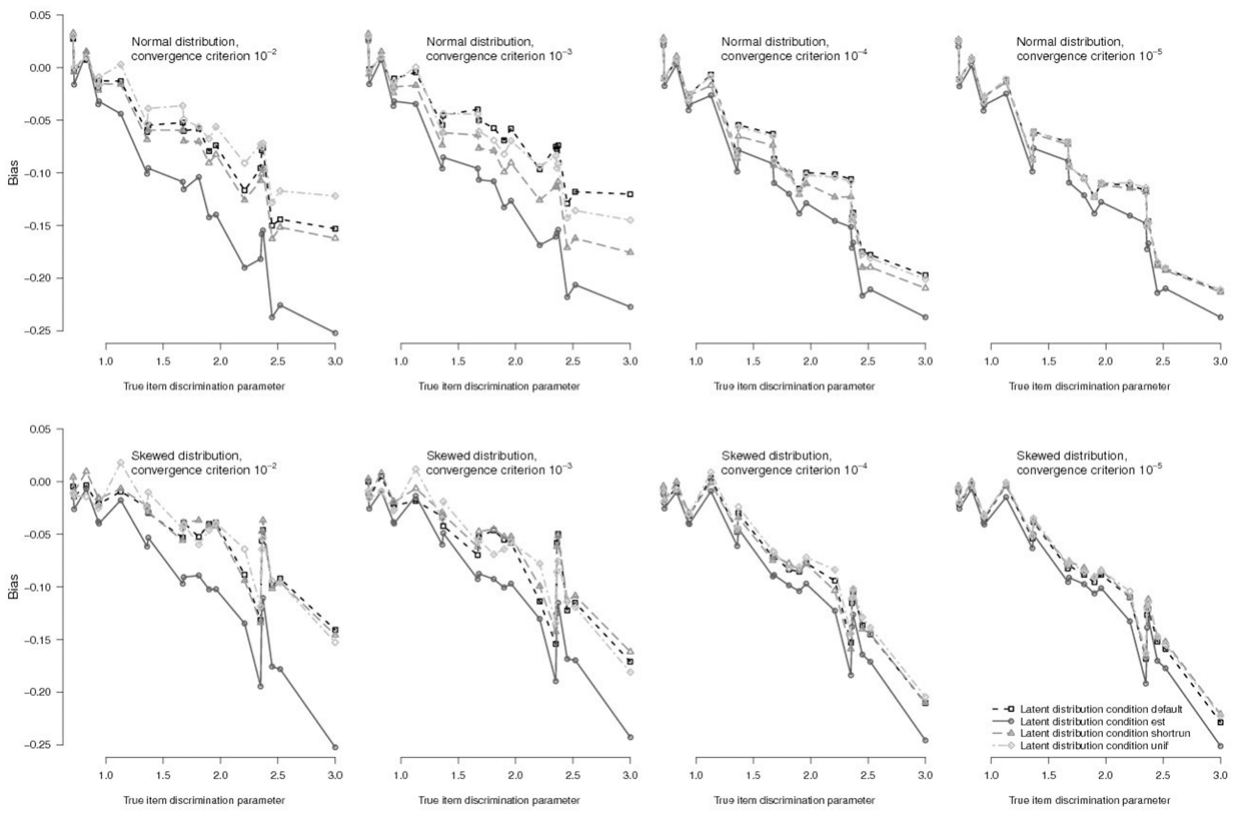
Bias for Discrimination Parameters of Single Items in EH Estimation for a Sample Size of *n* = 1000. Top panels depict bias for normal true latent distribution, bottom panels show bias for skewed true latent distributions, with tighter convergence criteria from left to right. Lines depict different initial value conditions for the estimated latent distribution. For clarity, initial values for item parameters are not depicted, as they hardly influenced results.

**Fig 3:**
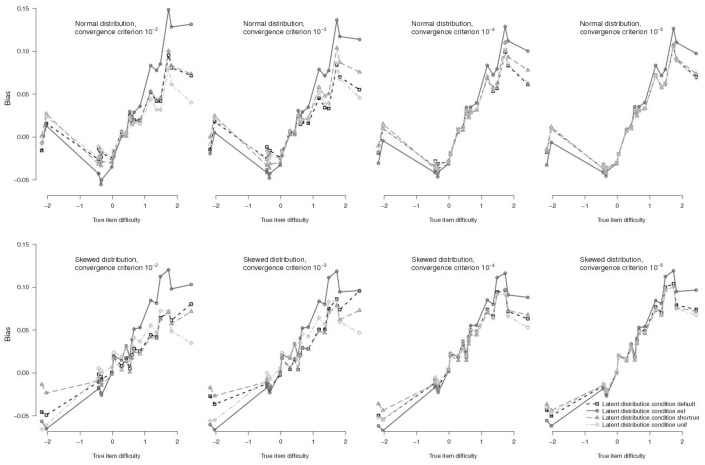
Bias for Difficulty Parameters of Single Items in EH Estimation for a Sample Size of *n* = 1000. Top panels depict bias for normal true latent distribution, bottom panels show bias for skewed true latent distributions, with tighter convergence criteria from left to right. Lines depict different initial value conditions for the estimated latent distribution. For clarity, initial values for item parameters are not depicted.

In regard to bias of single items, bias was most extreme for difficult items, as well as for items with a very high discrimination parameter. Relatedly, influences of initial values were also most notable for items with extreme difficulty (both very difficult and very easy items were affected by the choice of initial values), and for items with high discrimination parameters (Figs 2 and 3: lines of different initial value conditions diverge for extremer item parameters). For item discrimination parameters, the biggest difference in bias between different initial value conditions amounted to 0.193 for an item with discrimination parameter *a*_i_ = 3.00 (between conditions *true/unif* and *const/est* for *n* = 1000 and normal latent distribution, convergence criterion 10^−2^). For item difficulty parameter bias, the biggest difference was 0.150 for an item with difficulty parameter *b*_i_ = 2.43 (between the same conditions as above).

**Fig 4:**
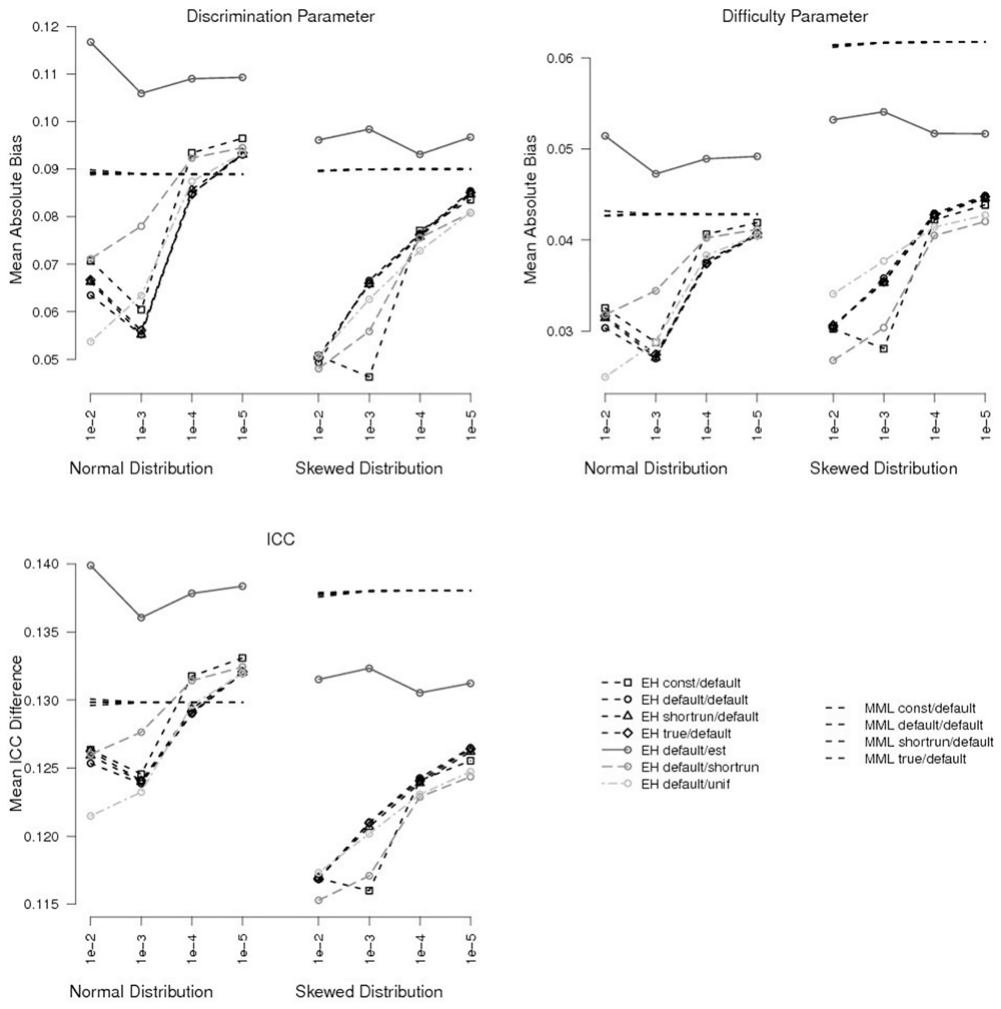
Test-level Bias and ICC Bias for Different Initial Values and Different Convergence Criterion Settings for a Sample Size of *n* = 1000. For clarity, only selected conditions are shown.

For tighter convergence criteria, the effects of initial values for the latent distribution decreased rapidly and were hardly noticeable for the tightest criterion (Fig 4, top, as well as Figs 2 and 3 for single items). They only explained 2.7% vs. 5.3% of variance of test-level bias in discrimination vs. difficulty parameters when using 10^−5^ as convergence criterion. In these conditions, initial values from the uniform distribution and from the shortrun procedure (conditions *unif* and *shortrun*) produced slightly lower bias than BILOG default values (condition *default*) when the true latent distribution was skewed.

When using standard MML estimation, there were virtually no effects of initial values on item parameters. They accounted for less than 0.01% vs. less than 0.1% of explained variance in discrimination vs. difficulty parameter bias for the default convergence criterion (Table 2), and even less for tighter criteria (results omitted for brevity; Figs 1 and 4 show that the different initial value conditions produce essentially the same results).

**Table 2:** Explained Variance for Bias of Item Discrimination and Item Difficulty Parameter for Standard MML Estimation with the Default Convergence Criterion.

		η^2^		
	*df*	Discrimination	Difficulty	ICC
Initial item parameters (*iip*)	3	.0000	.0005	.0000
Sample size (*n*)	2	.1296	.1854	.9609
True latent distribution (*tld*)	1	.5003	.4377	.0119
*iip* x *n*	6	.0000	.0000	.0000
*iip* x *tld*	3	.0001	.0001	.0000
*n* x *tld*	2	.3700	.3761	.0272
Residual	6	.0000	.0001	.0000

*Note*. See Table 1.

### 3.2 Effects of initial values on recovery of the latent distribution

Recovery of the true latent distribution was best when initial values from conditions *default* or *est* were used (lowest Earth Mover’s Distance). Other initial value conditions recovered the latent distribution rather poorly, especially the *shortrun* condition.

For tighter convergence criteria, recovery of the latent distribution got worse (i.e., increased values of Earth Mover’s Distance; Table 3). This effect was least severe when using condition *est* for initial values for the latent distribution. For the strictest convergence criterion, this condition exhibited by far the best recovery of the latent distribution. Of note, condition *est* showed highest bias in terms of item parameter recovery. Default initial values for the latent distribution (condition *default*) had second-lowest values for Earth Mover’s Distance, i.e., using default initial values was the second-best choice in terms of recovering the latent distribution.

**Table 3:** Recovery of Latent Distribution (Earth Mover’s Distance).

	Initial values for latent distribution			
Convergence criterion	default	est	shortrun	unif
Normal latent distribution				
10^−2^	0.130	0.186	0.517	0.368
10^−3^	0.366	0.238	0.621	0.541
10^−4^	0.674	0.297	0.738	0.728
10^−5^	0.720	0.308	0.772	0.772
Skewed latent distribution				
10^−2^	0.125	0.148	0.543	0.310
10^−3^	0.290	0.181	0.654	0.383
10^−4^	0.515	0.226	0.828	0.632
10^−5^	0.585	0.238	0.903	0.834

### 3.3 Effects of initial values on ICC recovery

Despite the effects of initial values on item parameters in the EH method, ICC recovery was hardly affected. Initial item parameters explained less then 0.1% of variance in ICC bias for the default convergence criterion and even less for tighter criteria (Table 1). Initial values for the latent distribution, which did influence item parameter estimation, did not affect ICC recovery (1.8% of explained variance for the default convergence criterion, less then 0.01% for the tightest criterion). The most important factor for ICC bias was sample size, explaining 94.1% of variance for the default convergence criterion and 96.0% for the tightest criterion (Fig 1, bottom).

### 3.4 Effects of the convergence criterion

Contrary to our expectations, results indicated that bias increased with tighter convergence criteria when estimating the latent distribution by means of the EH method. For *n* ≤ 500, lowest values of test-level bias were achieved with the loosest convergence criterion of 10^−2^, with BILOG default initial values (condition *default*) always among the best (only outperformed in medium samples with normal [true] latent distribution by initial values from a uniform distribution). For *n* = 1000, this pattern remained the same, but BILOG default values performed differently than other initial values. Bias decreased when the convergence criterion was reduced from 10^−2^ to 10^−3^, but increased for tighter criteria (Fig 4, top). Still, test-level bias was lowest for the loosest convergence criterion (with one exception for item discrimination parameter bias; Fig 4, top left). As reported above, initial values from a uniform distribution or from the shortrun procedure yielded the lowest values of bias for large samples.

Regarding single items, item discrimination parameter bias increased with tighter convergence criteria over the whole range of item discrimination, but most for items with high discriminatory power. In terms of item difficulty parameter, bias increased most for items with medium difficulty. A reduction of bias with tighter convergence criteria was only found for items with very high difficulty. However, this reduction was obviously not sizeable enough to reduce the test-level bias (as reported above). The fact that test-level bias increased with stricter convergence criteria might be related to the estimation of the latent distribution, which gets increasingly worse for tighter convergence criteria (as mentioned above; Table 3).

### 3.5 Effects of the true latent distribution

When using the EH method, bias was lower when the true distribution was skewed compared to when it was normal (Fig 1, top), particularly for the item discrimination parameter (between 13% and 17% of explained variance; Table 1). The differences between test-level bias for normal vs. skewed (true) latent distributions were largest for small samples and decreased with increasing sample size.

Results regarding the recovery of the latent distribution showed that the EH method was less efficient when the true latent distribution was normal compared to when it was skewed (mean Earth Mover’s Distance for normal vs. skewed true latent distributions = 0.500 vs. 0.462, Welch test *t*(37795.31) = 11.98, *p* < .001, Cohen *d* = 0.12). Furthermore, these differences were most severe for small samples (Cohen *d* = 0.15 vs. 0.14 vs. 0.12 for small vs. medium vs. large samples).

In regard to single items, bias was generally most severe for items with extreme difficulties (i.e., very easy or very difficult items). However, when comparing item difficulty bias between normal and skewed latent distribution, difficult items exhibited greater bias, while easy items exhibited smaller bias when the true latent distribution was normal (compared to when it was skewed). This pattern was more severe for looser convergence criteria (Fig 3). This finding might be related to the information available to estimate the item difficulties: With a positive skew, there is less information for easy items and more information for difficult items (compared to a normal distribution). Bias of difficulty parameters was indeed related to the density of the true latent distribution (*r* = -.63). It is also known that bias is related to the true item difficulty (scale expansion effect). In the present study, this effect amounted to *r* = .78. Bearing this in mind, item difficulty bias might best be explained by a linear model including both of these influences: This model explained 84% of the variance in item difficulty parameter bias, with all predictors being significant (greater bias was associated with lower density and higher difficulty; detailed results omitted for brevity).

When using standard MML estimation, bias values increased with increasing sample size when the true latent distribution was skewed (that is, when the assumed latent distribution was mis-specified; Fig 1). This interaction between sample size and true latent distribution accounted for 37.0% and 37.6% of explained variance for item discrimination and item difficulty parameters, respectively (Table 1).

Compared to the EH method, standard MML estimation results displayed comparable or even smaller bias for small samples, irrespective of the true latent distribution. Only in large samples, the EH method performed better than MML estimation. In other words, a mis-specified latent distribution seemed to be problematic only in larger samples.

## Discussion

This study examined the effects of initial values and of the convergence criterion for estimating parameters of the 2PL in BILOG-MG 3. In line with previous research [10], initial values for item parameters did not affect results notably. Initial values for the latent distribution, on the other hand, had noteworthy effects on bias of item discrimination and item difficulty parameters.

For the default convergence criterion 10^−2^, initial values for the latent distribution explained a considerable proportion of variance of item discrimination and item difficulty parameters. They explained more than half as much as sample size, which is known to be the most important factor for explaining bias in IRT (e.g., [21]). Conforming to prior research, a scale expansion effect was found (i.e., more bias for items with extreme difficulties; e.g., [4,21,22]). Relatedly, influences of initial values were more pronounced for very easy or very difficult items.

Initial values also affected the estimation of the latent distribution. Of note, initial values that allowed a better recovery of the latent distribution exhibited more item and test-level bias for difficulty as well as discrimination parameters. For stricter convergence criteria, condition *est* recovered the latent distribution best, but bias values for item parameters were highest.

Hence, choosing appropriate initial values for EM estimation seemed very important for parameter recovery when estimating the latent distribution by means of the EH method. BILOG-MG 3 default values seem to be a good choice, as these produced low values of bias in all conditions. Only with larger sample sizes, using initial values from a uniform distribution or from the shortrun procedure resulted in slightly lower values of bias for both item parameters. It seems likely that initial values generated by these methods are favorable with even larger samples. This result can be best understood from a Bayesian point of view, as using a uniform distribution as a prior distribution (i.e., initial values) may be seen as not introducing any prior information about the latent (posterior) distribution. Consequently, this approach seems best suited for large samples, because more information is available for estimating the latent distribution. For initial values from the shortrun procedure, a similar argument might apply: The short runs of the estimation procedure to generate initial values can be interpreted as an educated guess about suitable initial values, and this guess is also better when more information is available for the estimation process.

Effects of initial values were restricted to item parameters and the recovery of the latent distribution. Although the different strategies for initial value generation produced different results in terms of item parameter bias, ICC recovery was hardly affected by initial values. This is possibly related to a flat surface of the loglikelihood function, which has been found for single items in the 2PL [23]. A flat maximum might lead to dissimilar item parameters when approached from different directions in different initial value conditions. Furthermore, taking into account that parameters (weights) of the latent distribution and item parameters are estimated simultaneously in the EH method, a flat maximum of the likelihood function might also explain why initial value conditions with better recovery of the latent distribution exhibited higher bias for item parameters: Again, the maximum might be approached from different directions, leading to an impaired recovery of either the latent distribution of the item parameters.

Furthermore, differential effects of initial values on item parameters, ICCs, and the recovery of the latent distribution may also be related to properties of convexity (and non-convexity) of the involved optimization functions: Optimization with regards to the ICCs is conditional on a logistic function (Eq 1), and hence results in a convex optimization function, whereas this is not the case with regards to the optimization function of the latent distribution (we are thankful to the Academic Editors Lourens J. Waldorp and Thomas Niederkrotenthaler for pointing this issue out to us). Convex functions play an important role in optimization problems. Strictly convex functions have no more than one minimum, strictly concave functions (the negative of a convex function), which are of concern here, have no more than one maximum. This property, together with a flat likelihood, might explain why ICCs were hardly affected by the choice of initial values, even in the presence of biased item parameters. On the other hand, the existence of multiple local maxima may have affected the optimization with regards to the latent distribution. The better recovery of the latent distribution under condition *est* than under the other conditions in our simulation study is consistent with such a view: Only under this condition were initial values estimated from the data. This might have helped circumventing problems with multiple maxima in the optimization process as initial values were—on average—likely already closer to their optimal values under condition *est* than under the other conditions. The convexity and non-convexity of optimization functions needs to be considered in research on computational algorithms.

Regarding possible remedies to the effect of initial values on parameter estimates in IRT, a simple, albeit in the literature scarcely discussed, option exists: It is of paramount importance to set a sufficiently tight convergence criterion when estimating the latent distribution from data. Effects of initial values for the latent distribution diminished rapidly with tighter criteria and were hardly noticeable for the tightest criterion used in this study. This finding differs from results found for another method of estimating the latent distribution: In full non-parametric ML estimation [24], initial values for the latent distribution were found to affect item parameter bias even with strict convergence criteria [11]. This might be related to differences in the estimation algorithm, since this method also estimates the positions (and not only the weights) of the support points from data (hence, introducing more additional parameters that may be influenced by initial values). In other words, the EH method, as implemented in BILOG-MG 3, seems less sensitive to the choice of initial values when the convergence criterion is tight enough.

However, this remedy of tightening the convergence criterion entails a severe drawback when using the EH method in BILOG-MG 3. Despite minimizing the effects of initial values, our results showed that, contrary to what might be expected, item parameter bias increased for tighter criteria (irrespective of the initial values used). On average, this bias is slight, but nevertheless noteworthy, as users might involuntarily increase bias by tightening the convergence criterion, expecting to achieve just the opposite. Increase of bias was most pronounced in items of medium difficulty, and in items with high discrimination. Furthermore, recovery of the latent distribution got considerably worse when the convergence criterion was tightened. As item parameters and latent distribution are estimated simultaneously, this could have been caused by numerical inaccuracies in estimating the latent distribution, possibly due to insufficient information available in ranges of the latent ability distribution with few participants. These inaccuracies might have propagated and increased with the number of iterations, impairing the recovery of item parameters and of the latent distribution. Hence, this might be an explanation for increasing inaccuracy with tighter convergence criteria. To sum up, it cannot be advised to tighten the convergence criterion for practical application. Instead, it seems best to use the default convergence criterion when estimating the latent distribution from the data.

Furthermore, bias was found to be smaller when the true latent distribution was skewed (compared to when it was normal). With increasing sample size, this difference grew smaller. This might have been caused by general problems to estimate the latent distribution in small samples also reported in prior research [25].

Standard MML estimation of the 2PL, i.e., assuming (and not estimating) a standard normal latent distribution, was unaffected by the choice of initial item parameters. This result is in line with previously stated but unpublished results [10]. Moreover, MML estimation was found to have comparable or lower bias than the EH method, even when the latent distribution was mis-specified in small samples. This is in line with other research [25], which found standard MML to be preferable (in terms of better model fit) to the EH method for small sample sizes, even when the assumed latent distribution does not match the true latent distribution. This is caused by difficulties in estimating the latent distribution when information is sparse. The smaller number of parameters which have to be estimated in standard MML estimation possibly also contributes to this phenomenon. On the other hand, MML estimation was also outperformed by EH estimation when the true latent distribution was normal, although only in large samples, only for certain initial values, and only for loose convergence criteria. This might be related to the sampling variation in the simulation process, which inadvertedly causes the true latent ability distribution to deviate from a perfect normal distribution to some extent. The EH method is able to adapt to these deviations, therefore resulting in lower values for bias. As already elaborated above, the EH method is only beneficial if the sample is large, and if the convergence criterion is not too strict.

In summary, this study showed that BILOG-MG 3, a widely used software package to estimate parameters of the 2PL model, is only sensitive to initial values when estimating the latent distribution from data. Despite the fact that influences of initial values on item parameters decrease when using a tighter convergence criterion, this is not advisable in practice, as simultaneously item parameter bias increased when doing so. When estimating the latent distribution from data, it thus seems beneficial to retain the default convergence criterion of 10^−2^ and to use appropriate initial values. BILOG-MG 3 default values appeared favorable for small and medium samples, but estimating the latent distribution cannot be recommended in these cases altogether, because MML results were less biased even when the assumed latent distribution was mis-specified. In large samples, using initial values from a uniform distribution or from the shortrun procedure might be beneficial for reducing bias in item parameter estimates.
